# Meteorological drivers of hemorrhagic fever with renal syndrome in China’s Jiaodong Peninsula: an ecological time-series study from 2020 to 2024

**DOI:** 10.3389/fpubh.2025.1743845

**Published:** 2025-12-16

**Authors:** Xiaofang Guo, Ruixiao Li, Yan Li, Xueying Tian, Yanxin Gao, Lianlong Yu, Ti Liu, Qing Duan, Renpeng Li, Zengqiang Kou

**Affiliations:** 1 College of Public Health, Shandong Second Medical University, Weifang, China; 2 Shandong Center for Disease Control and Prevention, Jinan, China; 3 Shandong Provincial Key Laboratory of Intelligent Monitoring, Early Warning, Prevention and Control for Infectious Diseases, Jinan, China; 4 National Institute of Parasitic Diseases, Chinese Center for Disease Control and Prevention (Chinese Center for Tropical Diseases Research), Shanghai, China; 5 NHC Key Laboratory of Parasite and Vector Biology, Shanghai, China; 6 Shandong Provincial Center for Health Science & Technology and Talents Development, Jinan, China

**Keywords:** distributed lag non-linear model, exposure lag effect, generalized additive model, hemorrhagic fever with renal syndrome, meteorological factors

## Abstract

**Background:**

Hemorrhagic Fever with Renal Syndrome (HFRS) is a naturally occurring zoonotic disease significantly influenced by meteorological factors, with rodents serving as the primary reservoir. It imposes a substantial global disease burden. This study aims to investigate the non-linear and interactive effects of meteorological factors on HFRS, as well as the exposure-lag-response patterns on the Jiaodong Peninsula in China.

**Method:**

Daily meteorological data for the Jiaodong Peninsula from 2020 to 2024 were collected from the China Meteorological Data Sharing Service System. Daily incidence data for HFRS cases were collected from the China Disease Prevention and Control Information System. A generalized additive model with quasi-Poisson regression was conducted to examine the non-linear relationships and interactive effects between meteorological factors and HFRS. A distributed lag non-linear model was constructed to investigate the exposure-lag effects of meteorological factors on HFRS. Model analysis was conducted using R 4.5.1 software, and visualization was performed using ArcGIS 10.7 software.

**Result:**

From 2020 to 2024, a cumulative total of 1,121 cases of HFRS were reported in China’s Jiaodong Peninsula. Among these, Qingdao reported 594 cases, Yantai reported 438 cases, and Weihai reported 89 cases. HFRS exhibits a distinct seasonal pattern, with the peak incidence occurring annually from October to December. Spearman correlation analysis and random forest regression analysis were employed to screen the original meteorological factors. Ultimately, weekly average air pressure was excluded, while weekly average temperature, weekly average wind speed, weekly average humidity, and weekly average precipitation were incorporated into subsequent modeling. The results indicate that all four meteorological factors influence the occurrence of HFRS, exhibiting a pronounced non-linear relationship. Interaction analysis indicates that within an appropriate temperature range, increases in precipitation, relative humidity, and wind speed within certain thresholds can synergistically heighten the risk of HFRS incidence. Using the median meteorological factor as the baseline, the risk of HFRS significantly increased after a 13-week lag (RR = 1.185, 95% CI: 1.016–1.381) during periods of extremely high temperature (the 95th percentile of WAT, 27 °C). Under extremely low temperature (the 5th percentile of WAT, −1 °C), the risk of HFRS significantly increased after a 15-week lag period (RR = 1.189, 95% CI: 1.014–1.394). Under extremely high humidity (the 95th percentile of WAH, 88%), the risk of HFRS significantly increased after a 14-week lag period (RR = 1.262, 95% CI: 1.023–1.557). However, no statistically significant effect was observed at extremely low humidity (the 5th percentile of WAH, 50%).

**Conclusion:**

The prevalence of HFRS on China’s Jiaodong Peninsula exhibits a significant non-linear association with meteorological factors, accompanied by pronounced interaction effects and complex exposure lag effects. The findings of this study provide quantitative evidence for regional precision-based early warning and tiered prevention and control measures. Public health authorities should formulate disease prevention and control strategies based on these results to reduce the burden of HFRS.

## 1 Introduction

Hemorrhagic Fever with Renal Syndrome (HFRS), also known as epidemic hemorrhagic fever, is a naturally occurring zoonotic disease caused by the epidemic hemorrhagic fever virus (Hantavirus), with rodents as the primary reservoir. It is classified as a Category B infectious disease under the Law of the People’s Republic of China on the Prevention and Treatment of Infectious Diseases (1, 2). Rodents themselves do not develop the disease, but their secretions and excretions are rich in the virus. When dried, these materials readily form aerosols that can infect humans upon inhalation (3, 4). At the same time, certain domestic animals and bats have also been confirmed as potential sources of infection contributing to the spread of the virus (5). The clinical manifestations of HFRS are diverse, primarily including fever, hypotensive shock, headache, back pain, nausea, vomiting, loss of appetite, skin and mucosal hemorrhage, and acute kidney injury. Most patients exhibit laboratory findings of elevated white blood cell counts, decreased platelet counts, positive urine protein, and elevated serum creatinine or blood urea nitrogen levels (6, 7). Although HFRS is distributed worldwide, over 90% of cases are concentrated in Asia and Europe. China and South Korea are the primary high-incidence areas in Asia (8), while Russia and Finland are the primary high-incidence areas in Europe (9). Additionally, China has the highest number of HFRS cases globally, with cumulative reported cases accounting for over 70–90% of the world’s total reported cases (10). From a historical perspective, Shandong Province is one of the provinces in China most severely affected by the HFRS epidemic, with the number of cases accounting for approximately one-third of the total number of cases nationwide (11). In recent years, with the advancement of comprehensive prevention and control measures such as rodent control, vaccination, and environmental management (12, 13), the overall incidence of HFRS has shown a significant downward trend. However, the incidence rate remains high in some areas, and the affected geographical scope continues to expand. Therefore, the HFRS epidemic still warrants high attention (14).

As a typical zoonotic disease, the transmission dynamics of hemorrhagic fever with renal syndrome (HFRS) are profoundly influenced by the complex interactions of environmental determinants, with meteorological factors playing a particularly crucial role (15). Meteorological factors such as temperature, precipitation, and humidity directly influence the survival, reproduction, and distribution of rodent hosts, as well as the persistence and transmission of hantaviruses in the environment (16). Past epidemiological studies in China have confirmed this complex meteorological association from various perspectives. For example, Fang et al. (17) utilized panel data models to identify significant associations between precipitation, humidity, and temperature in Shandong Province and the incidence of HFRS. More in-depth studies have revealed the complex non-linear and hysteresis characteristics involved. Luo et al. (18) applied a distributed lag non-linear model (DLNM) to reveal a specific “danger range” for temperature affecting HFRS incidence and observed rainfall effects with delays lasting up to several weeks. Xue et al. (19) further indicated that short-term fluctuations and long-term changes in temperature exhibit distinct asymmetry in their impact on the incidence of HFRS. These findings collectively indicate that meteorological factors drive HFRS through a complex “exposure-lag-response” mechanism, far from a simple linear relationship.

This study utilizes monitoring data from China’s Jiaodong Peninsula to investigate the non-linear trends and interactive effects of meteorological factors on hemorrhagic fever with renal syndrome (HFRS) incidence using Generalized Additive Models (GAM) and Distributed Lag Non-linear Models (DLNM). Exposure-lag effects are also analyzed. The generalized additive model effectively controls for confounding variables by fitting the non-linear relationship between meteorological factors and disease through smoothing functions, thereby providing a scientific basis for public health early warning systems (20). The distributed lag non-linear model, as a classical statistical method, can simultaneously address the non-linear characteristics and lag effects in exposure-response relationships. Through its flexible modeling framework, it accurately quantifies the effects of complex exposures on health outcomes (21, 22). The Jiaodong Peninsula lies in the transition zone between humid subtropical and humid continental climates, possessing unique geographical and climatic conditions. Previous studies in this region have typically relied on traditional statistical methods, which may fail to adequately capture the complex non-linearities, interactive effects, and exposure lag effects that are characteristic of the relationship between weather and disease. The findings of this study are expected to provide deeper insights into the climate drivers of HFRS transmission and offer scientific basis for developing targeted early warning systems and intervention strategies in this high-risk region.

## 2 Materials and methods

### 2.1 Study area

**Figure 1:**
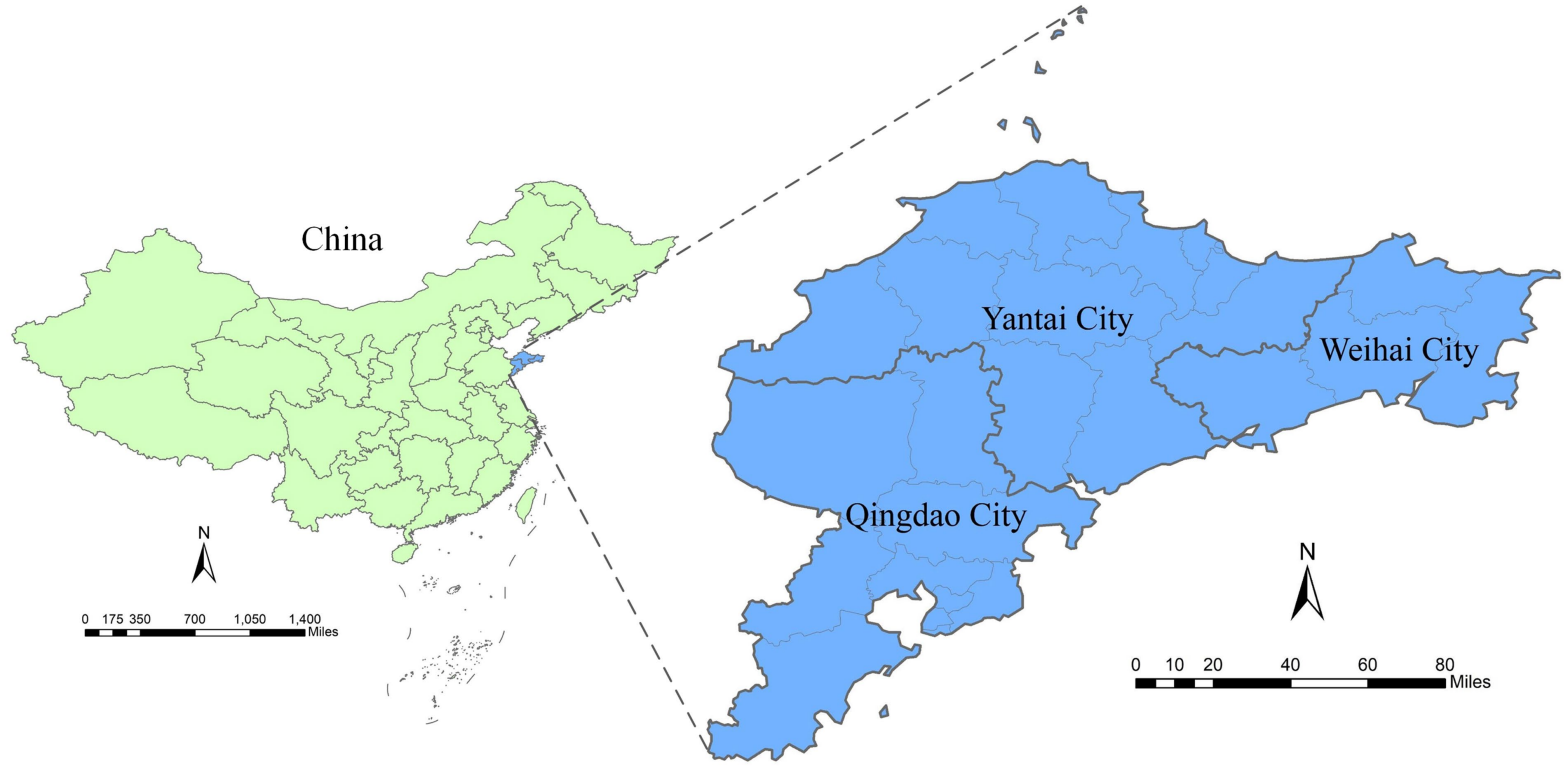
Geographic location of China’s Jiaodong Peninsula.

The Jiaodong Peninsula encompasses the three prefecture-level cities of Qingdao, Yantai, and Weihai in Shandong Province. Located in eastern China, it is the country’s largest peninsula. Named “Jiaodong” for its shape resembling a horn dipping into the sea, as it is surrounded by the ocean on three sides. Its longest east–west dimension is 290 kilometers, with a maximum north–south width of 190 km. The total area is approximately 26,600 square kilometers, and the total permanent resident population is 20,392,300. The Jiaodong Peninsula spans longitudes 119°30′E to 122°42′E and latitudes 35°05′N to 38°23′N, extending across subtropical and humid continental zones. It features a warm climate characterized by dry winters and hot summers (23) (Figure 1).

### 2.2 Data source

All cases of hemorrhagic fever with renal syndrome reported in the Jiaodong Peninsula (Qingdao City, Yantai City, and Weihai City) from January 1, 2020, to December 31, 2024, were collected from the China Information System for Disease Control and Prevention (CISDCP). This includes confirmed cases, suspected cases, and clinically diagnosed cases. From the China Meteorological Data Sharing Service System^1^, daily meteorological data were collected for the period from January 1 to December 31, 2020, from 24 meteorological stations across the Jiaodong Peninsula. The data include air temperature, atmospheric pressure, relative humidity, wind speed, and precipitation. The meteorological data from each site within every prefecture-level city is averaged to represent the regional weather conditions. Initially obtained at the daily scale, the data is aggregated to the weekly scale through computational processing. Additionally, missing values in the data are handled using multiple imputation methods from the R package “mice.” Weekly summaries of collected cases of hemorrhagic fever with renal syndrome and meteorological data were compiled to establish a basic database.

### 2.3 Research methods

#### 2.3.1 Variable filtering

By combining random forest regression analysis and Spearman correlation analysis, the final variables for subsequent modeling were selected. The random forest regression model was employed to assess the importance of various meteorological variables in HFRS incidence. When setting parameters, the random forest regression process is first run 10 times, and the average of each variable’s relative importance score is selected as the basis for evaluation. After excluding variables that are highly correlated with others but have low intrinsic importance, a new random forest regression analysis was conducted to systematically evaluate variable importance (24). Spearman correlation analysis is used to assess the correlation between various meteorological variables and determine whether they exhibit collinearity. If two variables exhibit high correlation (*r* > 0.7), only those variables with higher importance in the random forest regression analysis are retained, thereby effectively mitigating multicollinearity issues (25).

#### 2.3.2 Generalized additive model

A generalized additive model was employed to assess the non-linear relationships and interaction effects between HFRS incidence and meteorological variables (26). Since the incidence of infectious diseases typically exhibits excessive dispersion, it is assumed that the distribution of HFRS cases follows a quasi-Poisson distribution (27). Based on this, a GAM model was selected to model the relationship between meteorological variables and the log-expectation of case counts. Using the logarithmic expected value of weekly HFRS incidence as the dependent variable and core meteorological factors as independent variables, this study employs smoothing functions to investigate the non-linear relationship between meteorological variables and disease risk. Simultaneously, to calibrate the model, the time trend was treated as a confounding factor, and the degrees of freedom (df) for the spline functions of each meteorological variable were estimated using generalized cross-validation (GCV). Select the combination of degrees of freedom that minimizes the GCV value to balance model fit and complexity. The formula for this model is:


$$\log(Y_{t})=\alpha +s({\mathrm{MF}}_{t},\mathrm{df})+s(\text{Week},\mathrm{df})$$

Where Log(*Y*_t_) represents the logarithmic transformation of the weekly HFRS case count, α denotes the intercept, MF_t_ is the meteorological factors for the current week, s() indicates the penalized spline function, Week refers to the week in which HFRS cases occurred, and df is the degrees of freedom.

#### 2.3.3 Distributed lag non-linear model

Meteorological factors typically exert a lagged effect on infectious diseases (28, 29). The GAM model can only analyze impacts within specific periods and cannot account for lagged characteristics under continuous exposure levels spanning multiple weeks, making it prone to multicollinearity. The DLNM model combines generalized additive models (GAM) with distributed lag non-linear models (DLNM). By incorporating a lag dimension into the exposure-response relationship through cross-basis functions, it simultaneously evaluates both delayed exposure effects and non-linear effects. This study employed a DLNM model to investigate the exposure-lag effects of meteorological variables, calculating relative risks (RR) to assess the impact of meteorological factors at different lag periods on the incidence of HFRS. By using a meta-analysis to combine DLNM model parameters from various regions, the exposure-lag curve for the Jiaodong Peninsula was ultimately obtained. To mitigate the effects of excessive dispersion, a quasi-Poisson distribution model with a log-link function is employed when setting parameters. Simultaneously, the robustness of the model was assessed through sensitivity analysis (30). The formula for this model is:


$$\text{logE}(Y_{t})=\alpha +\mathrm{cb}(M,{\mathrm{df}}_{1})+\mathrm{ns}({\mathrm{cov}}_{t},{\mathrm{df}}_{2})+\mathrm{ns}(\text{time},{\mathrm{df}}_{3})$$

Where *E*(Yt) denotes the weekly HFRS case count for week t, α is the intercept, cb represents the cross-term basis function for meteorological variables, M denotes the weekly mean temperature, time is the time variable controlling for seasonality and long-term trends, ns is the natural spline function, and Cov is the covariate variable controlling for the influence of other meteorological factors on week t. This study set the maximum lag period to 15 weeks (31, 32).

#### 2.3.4 Statistical analysis

Using descriptive research to analyze the basic characteristics of HFRS and meteorological factors (air temperature, atmospheric pressure, wind speed, relative humidity, precipitation) in the Jiaodong Peninsula from 2020 to 2024. Use ArcGIS software (version 10.7) to create distribution maps. Employ R software (version 4.5.1) with packages such as “randomForest,” “dlnm,” “mgcv.,” and “ggplot2” to construct GAM and DLNM models. The confidence intervals (CI) of all two-side statistical tests was set as 95%. A result is considered statistically significant if the confidence interval does not include 1.

## 3 Results

### 3.1 Basic information

From 2020 to 2024, the Jiaodong Peninsula reported a cumulative total of 1,121 cases of HFRS. Among these, Qingdao reported 594 cases, Yantai reported 438 cases, and Weihai reported 89 cases. HFRS exhibits distinct seasonal patterns, with the peak incidence occurring annually from October to December. The weekly averages for atmospheric pressure, air temperature, relative humidity, precipitation, wind speed, and number of cases were 1,010 hPa, 13.59 °C, 68.60%, 2.50 mm, 2.99 m/s, and 1.43 cases, respectively (Table 1). The temporal distribution of HFRS cases and meteorological factors (Supplementary Figure 1).

**Table 1:** Basic information on HFRS cases and meteorological factors in the Jiaodong Peninsula, China, 2020–2024.

Variables	Mean	Min	P25	P50	P75	Max
WAAP (hPa)	1,010	991	1,002	1,010	1,017	1,028
WAT (°C)	13.59	−6.47	4.56	13.95	22.47	29.18
WAH (%)	68.60	33.93	59.76	68.12	77.58	93.27
WAP (mm)	2.50	0	0.05	0.72	3.20	31.17
WAWS (m/s)	2.99	1.64	2.54	2.96	3.40	5.39
Cases	1.43	0	0	1	2	19

Mean denotes the average value, Min denotes the minimum value, P25 denotes the 25th percentile, P50 denotes the 50th percentile, P75 denotes the 75th percentile, Max denotes the maximum value, WAAP stands for Weekly Average Air Pressure, WAT denotes Weekly Average Temperature, WAH denotes Weekly Average Humidity, WAP denotes Weekly Average Precipitation, WAWS denotes Weekly Average Wind Speed, and cases denotes the number of weekly cases.

### 3.2 Correlation and importance analysis of HFRS and meteorological factors

**Figure 2:**
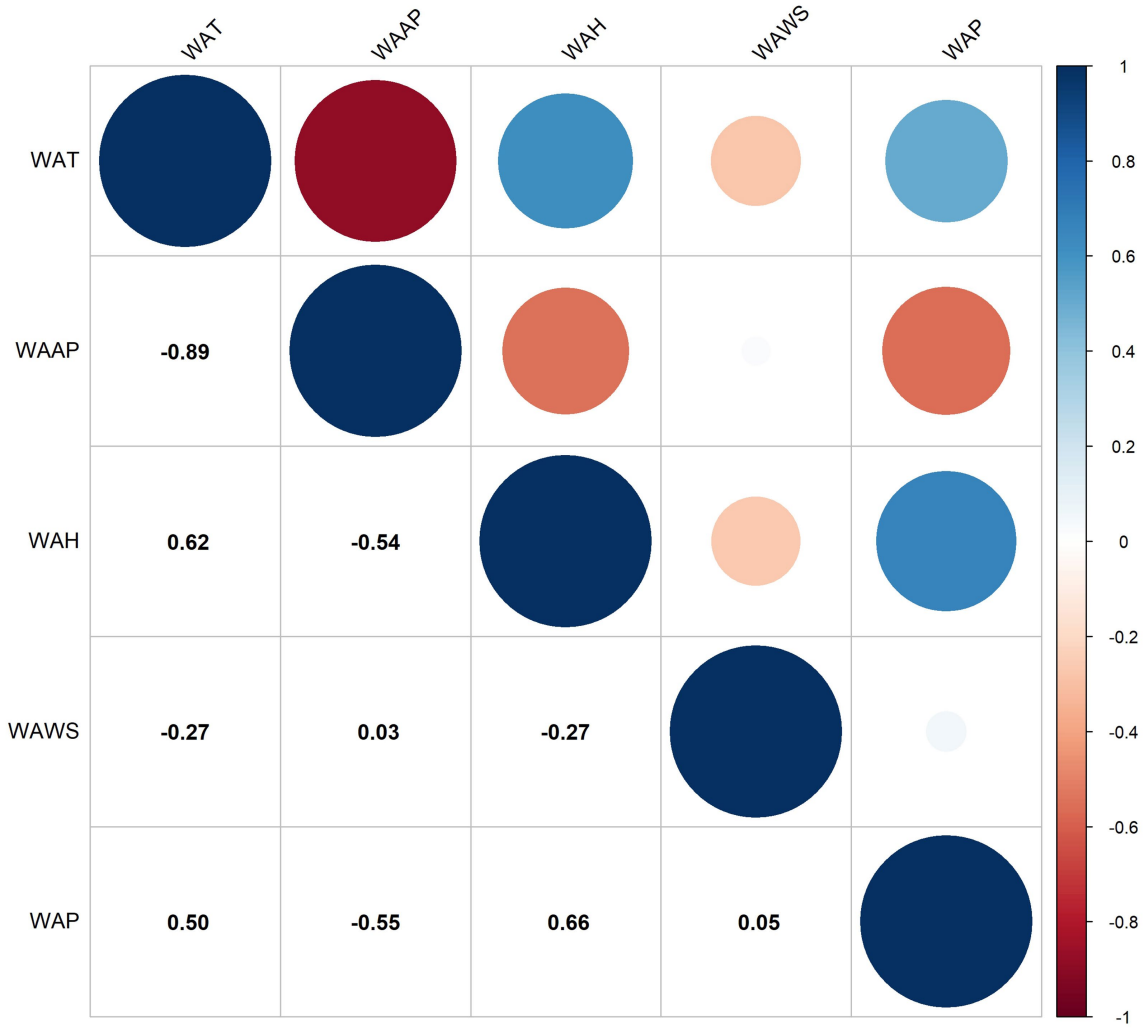
Spearman correlation analysis of meteorological factors, 2020–2024. WAAP denotes weekly average air pressure, WAT denotes weekly average temperature, WAH denotes weekly average humidity, WAP denotes weekly average precipitation, and WAWS denotes weekly average wind speed.

**Figure 3:**
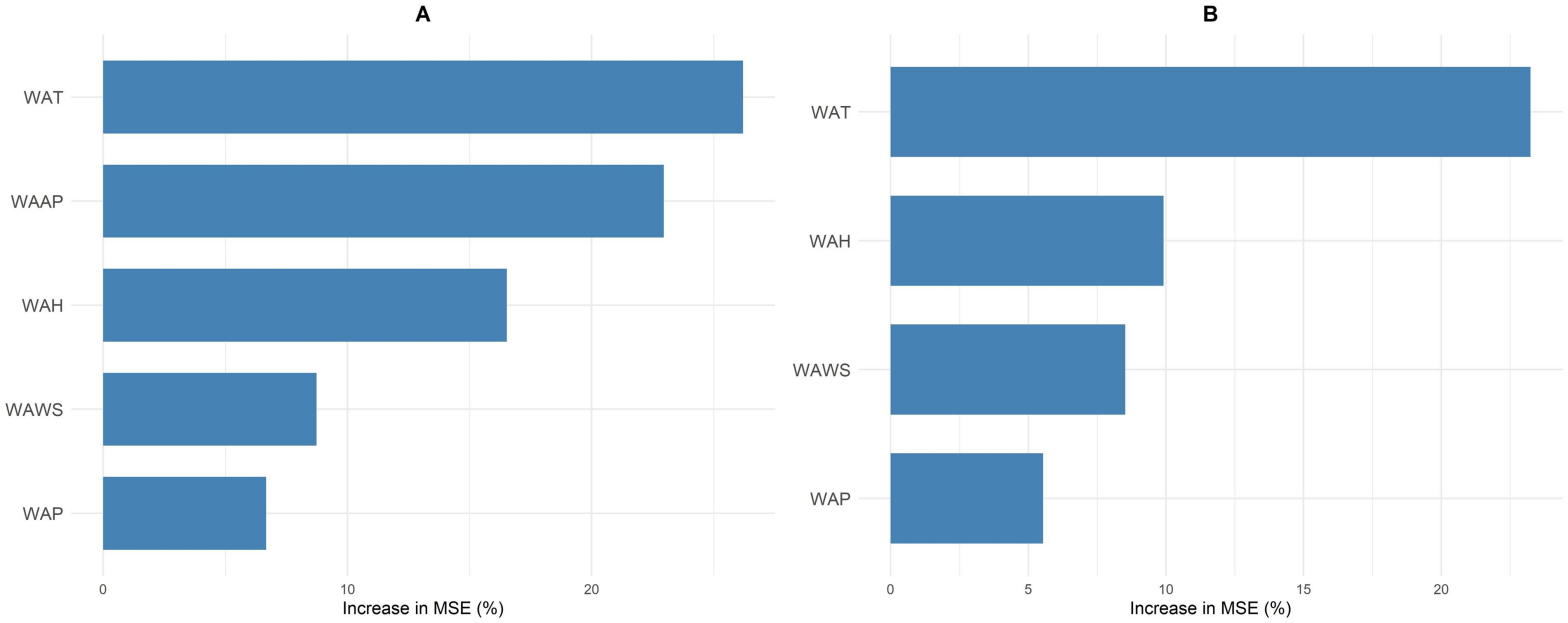
Ranking of meteorological factors by importance for HFRS, 2020–2024. **(A)** Initial ranking of meteorological factors by importance. **(B)** Final ranking of meteorological factors after screening.

Spearman correlation analysis revealed a positive correlation between weekly average air pressure and weekly HFRS case counts. Conversely, weekly average temperature, weekly average wind speed, weekly average humidity, and weekly average precipitation exhibited negative correlations with weekly HFRS case counts. The correlation between weekly average temperature and weekly average air pressure exceeded 0.7, while correlations among the remaining variables all fell below 0.7 (Figure 2). Analysis of the random forest regression model indicates that meteorological factors are ranked in order of importance as follows: weekly average temperature, weekly average air pressure, weekly average humidity, weekly average wind speed, and weekly average precipitation (Figure 3A). Based on the results of Spearman’s correlation analysis and random forest regression analysis, weekly average air pressure was ultimately excluded from subsequent modeling. Using the random forest regression model again, the importance ranking was as follows: weekly average temperature, weekly average humidity, weekly average wind speed, and weekly average precipitation (Figure 3B).

### 3.3 Correlation analysis between HFRS and meteorological factors

**Figure 4:**
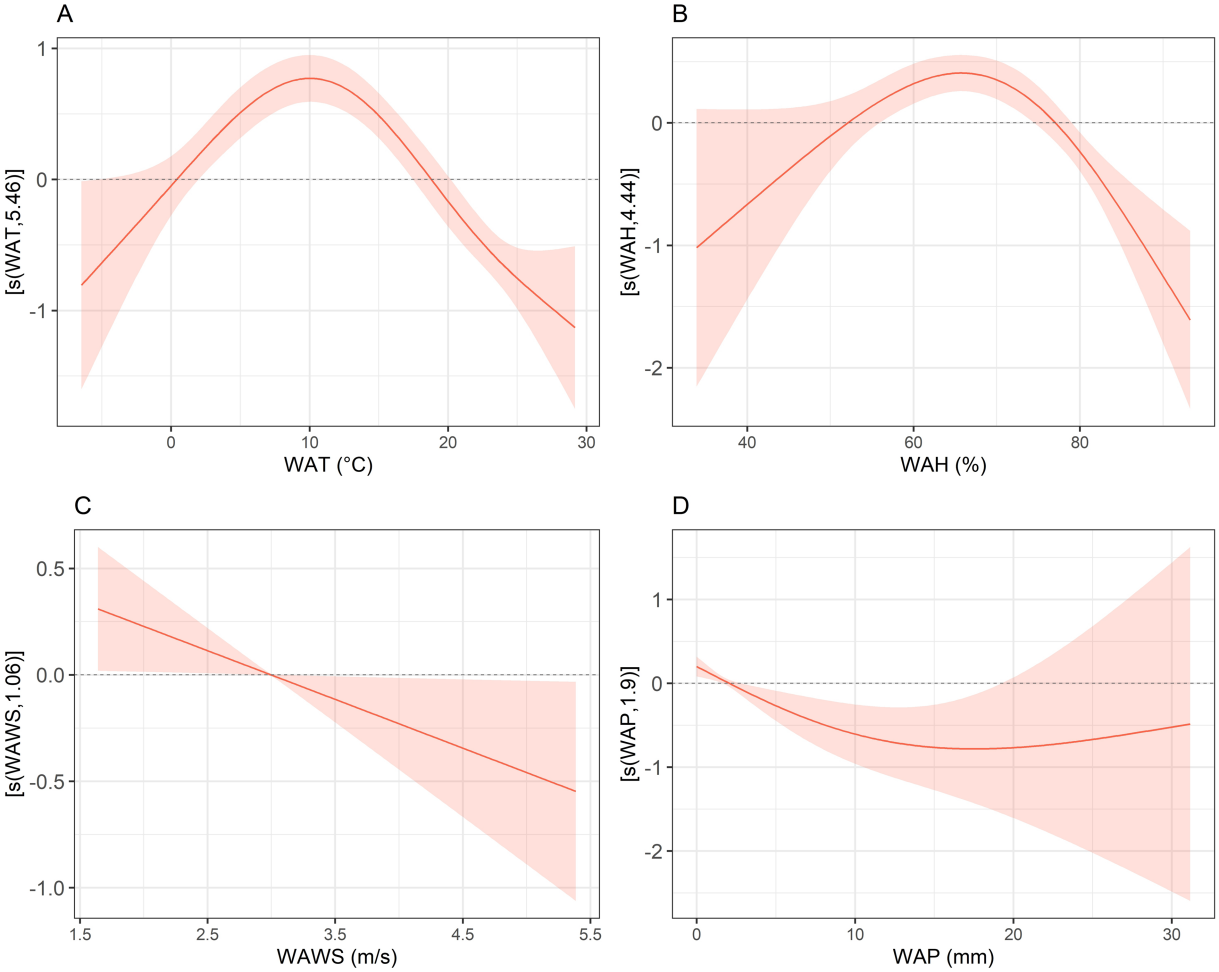
Univariate analysis of meteorological factors on HFRS incidence risk in the Jiaodong Peninsula, 2020–2024. **(A)** Univariate analysis of WAT on HFRS. **(B)** Univariate analysis of WAH on HFRS. **(C)** Univariate analysis of WAWS on HFRS. **(D)** Univariate analysis of WAP on HFRS.

**Figure 5:**
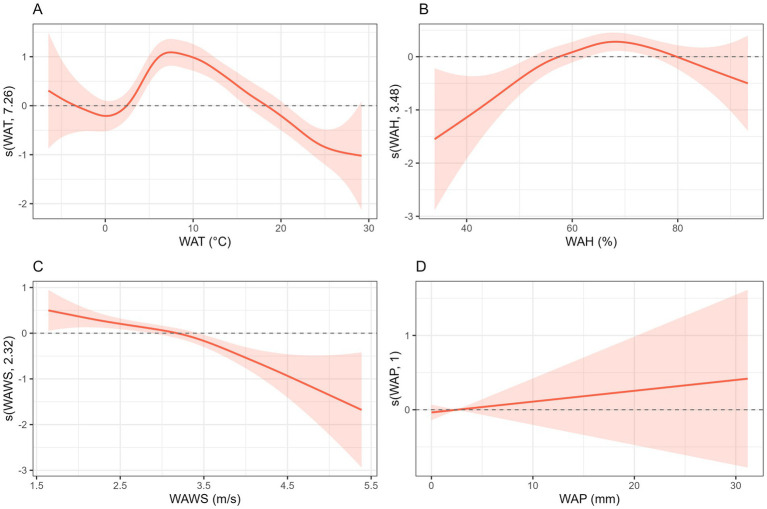
Multivariate analysis of meteorological factors on HFRS incidence risk in the Jiaodong Peninsula, 2020–2024. **(A)** Multivariate analysis of WAT on HFRS. **(B)** Multivariate analysis of WAH on HFRS. **(C)** Multivariate analysis of WAWS on HFRS. **(D)** Multivariate analysis of WAP on HFRS.

First, a generalized additive model (GAM) was employed to conduct univariate analyses of the incidence of HFRS in relation to various meteorological factors. Results indicate that when the weekly average temperature falls below 10 °C, the risk of HFRS increases with rising temperatures. When the weekly average humidity falls below 70%, the risk of HFRS increases with rising humidity. As the weekly average wind speed increases, the risk of HFRS incidence decreases. As weekly average precipitation increases, its impact on HFRS disease follows a U-shaped curve, exhibiting a non-linear trend that first decreases and then increases (Figure 4). A multivariate analysis was conducted using the GAM model to further examine the relationship between the number of HFRS cases and various meteorological factors. Results indicate that when the weekly average temperature ranges from approximately 0 to 7 °C, the risk of HFRS increases with rising temperatures. When the weekly average humidity is below 70%, the risk of HFRS increases with rising humidity. As the weekly average wind speed increases, the risk of HFRS incidence decreases. As weekly average precipitation increases, the risk of HFRS incidence rises (Figure 5).

### 3.4 Interaction analysis of meteorological factors on the risk of HFRS onset

**Figure 6:**
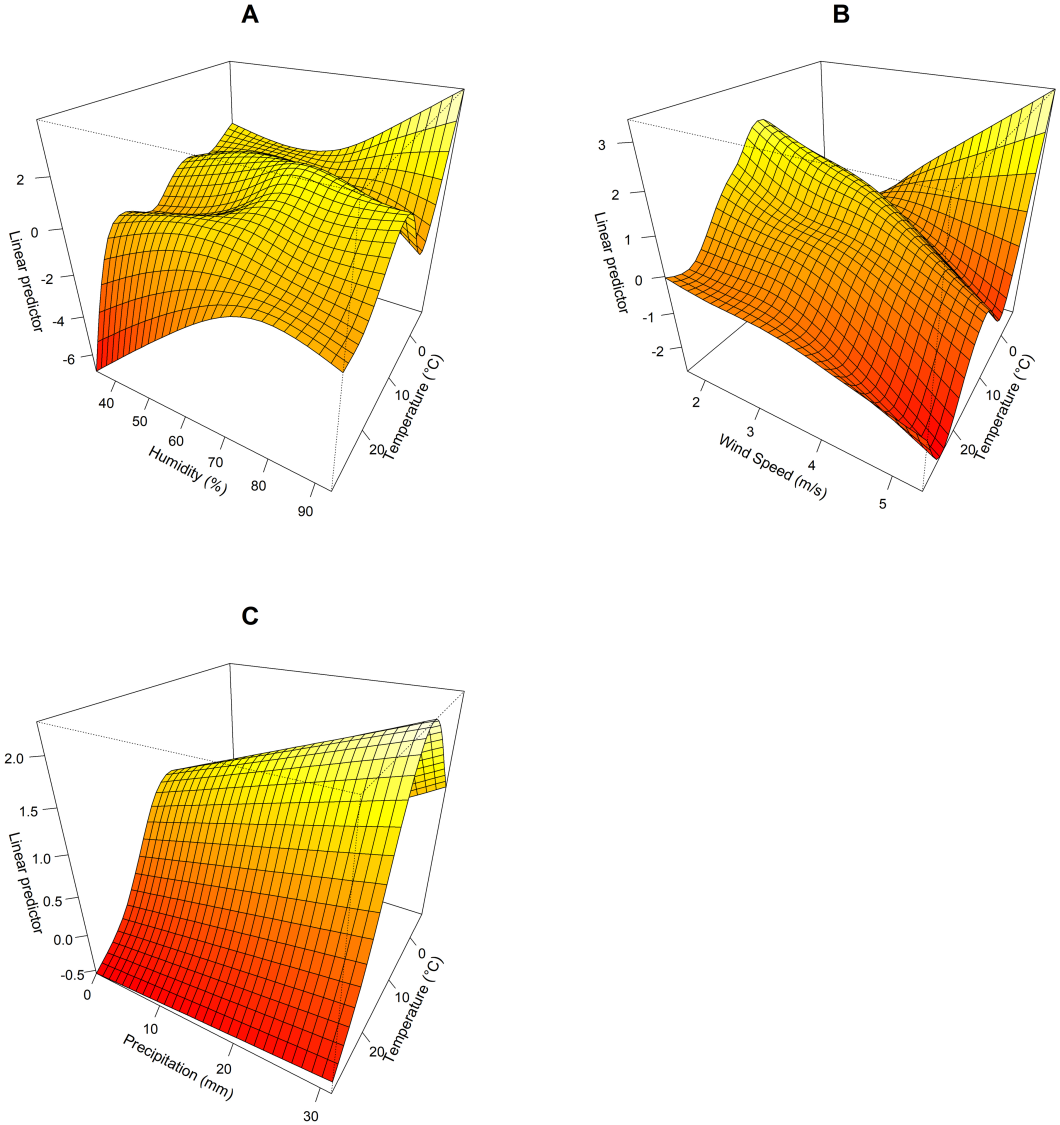
Three-dimensional interaction map of different meteorological factors in the Jiaodong Peninsula, 2020–2024. **(A)** The interactive effects of temperature and humidity on HFRS. **(B)** The interactive effects of temperature and wind speed on HFRS. **(C)** The interactive effects of temperature and precipitation on HFRS.

A three-dimensional interaction map was constructed to visualize the effects of various meteorological factors on HFRS incidence (Figure 6). The results revealed significant interactions among weekly average temperature, weekly average wind speed, weekly average humidity, and weekly average precipitation, each exerting varying degrees of influence on the risk of HFRS occurrence. When temperatures remain constant and wind speeds and humidity levels fall within specific ranges, the risk of HFRS increases as both wind speed and humidity rise (Figures 6A,B). If both temperature and precipitation increase simultaneously, they will produce a significant synergistic effect on the risk of HFRS (Figure 6C).

### 3.5 The lagged effect of meteorological factors on HFRS incidence

**Figure 7:**
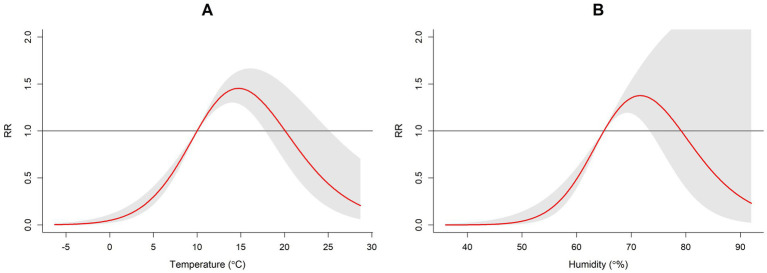
Exposure response curve diagram of meteorological actors and HFRS on the Jiaodong Peninsula, 2020–2024. **(A)** Temperature-exposure response curve for HFRS. **(B)** Humidity-exposure response curve for HFRS.

To further evaluate the non-linear relationship between weekly average temperature and weekly average humidity with exposure lag effects, three-dimensional interaction maps, contour plots, and lag distribution plots were constructed using the median values of meteorological factors as reference points. When temperatures range between 10–20 °C, the risk of HFRS is relatively high. Above 20 °C, the relative risk of HFRS decreases as temperatures rise (Figure 7A). At humidity levels between 65 and 80%, the likelihood of HFRS increases. When humidity exceeds 80%, the relative risk of HFRS decreases as humidity rises (Figure 7B).

**Figure 8:**
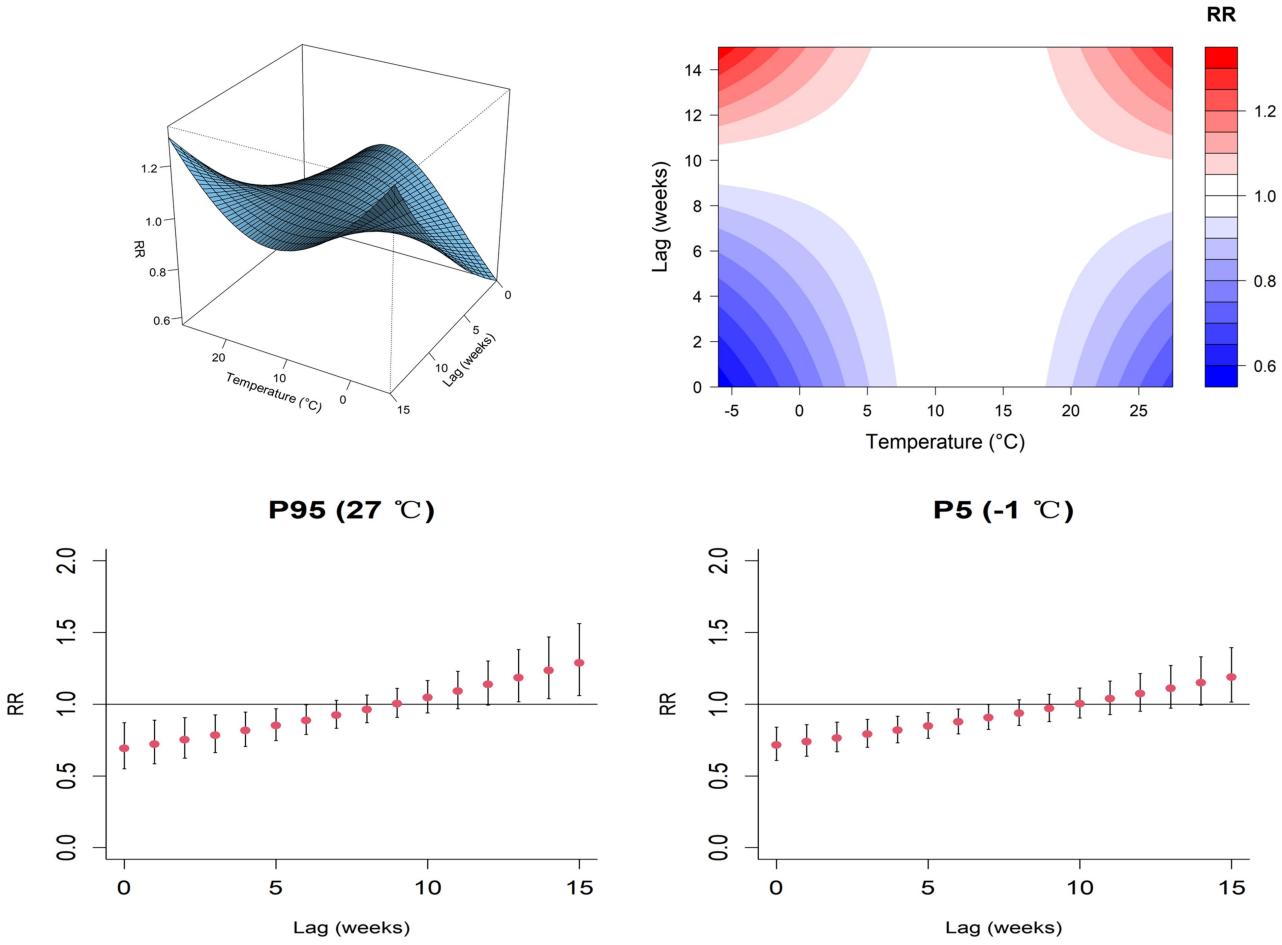
Weekly average temperature and exposure lag effects of HFRS in the Jiaodong Peninsula, 2020–2024.

By using the 95th percentile and 5th percentile of weekly average temperature and humidity, calculate the relative risk and 95% confidence interval for HFRS incidence at different lag weeks. Under extremely high temperature (95th percentile of weekly average temperature, 27 °C), a 6-week lag was associated with a reduced risk of HFRS occurrence (RR = 0.887, 95% CI: 0.790–0.995). After a 13-week lag period (RR = 1.185, 95% CI: 1.016–1.381), the risk of developing HFRS significantly increased. Under extremely low temperature (the 5th percentile of weekly average temperatures, −1 °C), a 7-week lag was associated with a reduced risk of HFRS occurrence (RR = 0.906, 95% CI: 0.824–0.997). After a 15-week lag period (RR = 1.189, 95% CI: 1.014–1.394), the risk of developing HFRS significantly increased (Figure 8).

**Figure 9:**
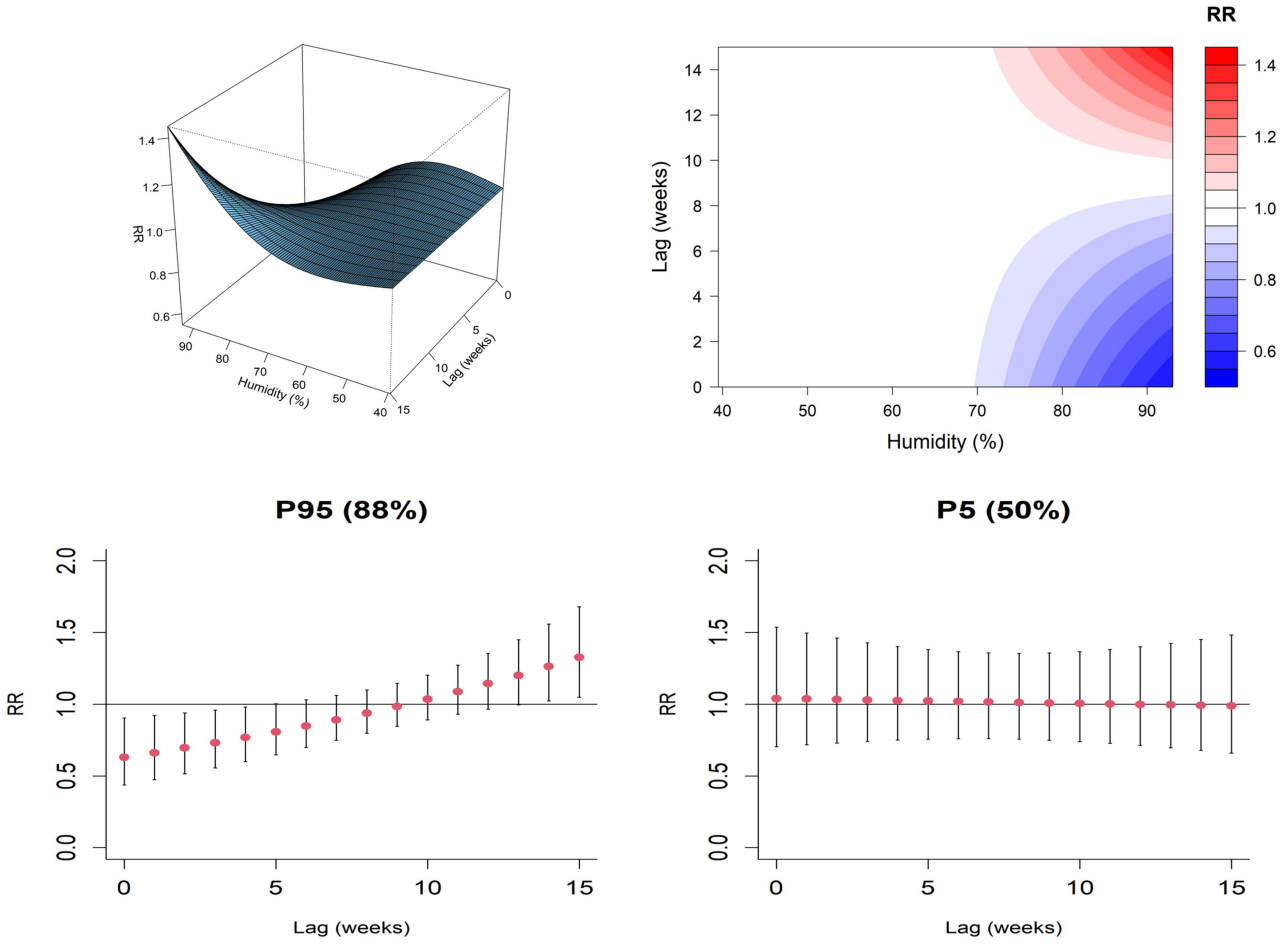
Weekly average humidity and exposure lag effects of HFRS in the Jiaodong Peninsula, 2020–2024.

Under extremely high humidity (95th percentile of weekly average humidity, 88%), a 4-week lag was associated with a reduced risk of HFRS occurrence (RR = 0.768, 95% CI: 0.601–0.981). while a significant increase in HFRS incidence risk was observed 14 weeks later (RR = 1.262, 95% CI: 1.023–1.557). Under extremely low humidity (the 5th percentile of weekly average humidity, 50%), the 95% confidence interval for the relative risk (RR) included 1, indicating that the effect estimate was not statistically significant (Figure 9). In the distributed lag non-linear model, sensitivity analysis conducted by adjusting the degree of freedom of natural spline functions yielded non-linear relationships that largely aligned with the exposure-lag effects and the main model trend, indicating robust reliability of the model.

## 4 Discussion

Hemorrhagic fever with renal syndrome (HFRS), as a typical naturally occurring zoonotic disease, poses a serious threat to public health. This study utilized HFRS surveillance data and meteorological records from the Jiaodong Peninsula in China between 2020 and 2024 to construct a Generalized Additive Model (GAM) and a Distributed Lag Non-linear Model (DLNM). It systematically examined the association between meteorological factors and the number of HFRS cases on the Jiaodong Peninsula in China. This study provides new insights into the climate-driven mechanisms underlying HFRS transmission, offering a scientific basis for epidemic prevention and control in the region. Research findings indicate that the influence of meteorological factors on HFRS does not follow a simple linear relationship but exhibits a complex interaction pattern (17). Among these, the importance of the weekly average temperature is most prominent. At the same time, there exists an interactive synergistic effect between weekly average temperature and other meteorological factors, which collectively influence the occurrence of HFRS. Weekly average temperature and weekly average humidity both exhibit significant non-linear relationships and exposure lag effects on the risk of HFRS (33–35).

Previous studies have indicated that temperature is one of the key drivers promoting HFRS transmission, with a complex non-linear relationship existing between the two (36). Both low and high temperatures can directly or indirectly affect the survival, reproduction, and distribution of rodents, as well as the survival and transmission of hantaviruses in the environment (37, 38). For example, Tian and Stenseth (39) research indicates that temperature can influence HFRS transmission through multiple pathways, including affecting rodent survival rates and population density, as well as the intensity of human outdoor activities. In this study, the results of the random forest regression model indicate that weekly average temperature is the most significant factor influencing the risk of HFRS incidence on the Jiaodong Peninsula. Its exposure-response relationship exhibits an approximate inverted U-shaped curve, consistent with findings from previous epidemiological studies on HFRS (11, 33). The risk of HFRS infection is highest when temperatures range between 10 and 20 °C. This temperature range corresponds to autumn in China’s Jiaodong Peninsula, aligning with the peak incidence period (12, 35) and consistent with the finding in this study that most HFRS cases occurred between October and December. Further analysis revealed that the lag effect in HFRS incidence exhibited significant variations depending on temperature levels and the number of lagging weeks. Under conditions of extremely high temperature or extremely low temperature, the risk of HFRS infection is significantly reduced, and the lag effect period is prolonged. Extremely high temperature significantly increases the risk of HFRS onset after a 13-week lag period (RR > 1, 95% CI does not include 1). Following a 15-week lag period, the risk of developing HFRS significantly increases during periods of extremely low temperature. This may be because extremely high temperature initially suppresses the survival and reproduction of rodents, causing their population density to plummet in the short term. Simultaneously, Hantaviruses are easily inactivated at high temperatures, thereby reducing opportunities for human exposure to the virus. During the late stages of extremely high temperature (typically corresponding to autumn), improved survival conditions lead rodents to enter a peak breeding period, thereby increasing the risk of HFRS outbreaks (18). The delayed emergence of extreme cold’s lag effect on HFRS may be explained as follows: Initial low temperatures suppressed activity frequency and population reproduction among host rodents (such as the black-striped field mouse), reducing viral transmission opportunities (40). Following a prolonged lag period, rodents migrated toward human settlements due to survival pressures, thereby increasing human-rodent contact rates. Additionally, prolonged exposure to low temperatures may prolong the survival time of viruses in excretions, enhancing their stability and infectivity. Combined with inadequate indoor ventilation during cold seasons, which increases the risk of aerosol exposure, these factors collectively elevate the risk of HFRS outbreaks (3, 4).

The effect of humidity on HFRS exhibits a lag-dependent interactive effect. Extremely high humidity may reduce the risk of HFRS onset in the short term (Early lag period) (31). This conclusion aligns with the findings of Sun et al. (31), where the confidence interval for high humidity only became statistically significant in the late latent period, indicating a higher relative risk of HFRS occurrence. One possible explanation is that short-term extreme humidity restricted rodent activity ranges, causing them to seek higher ground and more concealed habitats, thereby reducing human-rodent contact. The heavy moisture in the air increased the weight of aerosol particles, inhibiting viral “aerosol transmission” and thus blocking short-term infection pathways. Concurrently, altered human activity patterns reduced outdoor exposure risks (41). During the mid-to-late stages of the lag period, rodents and viruses gradually adapted to the humid environment, and the protective effect of extreme humidity diminished (RR > 1). High humidity promotes vegetation growth and increases food resources, favoring an increase in rodent population density. Additionally, viruses in rodent excrement can survive for 4–6 weeks in moist environments (2–3 times longer than in drier conditions) (42, 43). The mid-to-late lag period typically corresponds to the warm-temperate rainy season on China’s Jiaodong Peninsula. This aligns with ecological characteristics such as expanded rodent habitats (e.g., increased soil moisture in farmlands and orchards) and viral dissemination via rainfall (e.g., broader contamination from excrement) (31). The prevalence of HFRS across different lag periods is influenced not only by meteorological factors but also by factors such as hantavirus serotypes, increased human outdoor activities in spring and autumn, seasonal fluctuations in rodent populations, and rodent density (44). The specific mechanisms underlying these relationships warrant further investigation.

The interaction analysis in this study indicates that various meteorological factors exert complex interactive synergy effect on the incidence of HFRS (18, 34). When weekly average wind speed, weekly average precipitation, and weekly average humidity fall within different ranges, their respective effects on weekly average temperature vary. Within the suitable temperature range, increases in precipitation and relative humidity within certain thresholds significantly elevate the risk of HFRS outbreaks. This aligns with the habitat requirements of HFRS reservoir animals—relatively humid environments and abundant water sources provide ideal conditions for rodent survival and reproduction, thereby increasing the risk of viral transmission (31). Similarly, within suitable ranges, increases in temperature and wind speed also elevate the risk of HFRS outbreaks. This may be because higher wind speeds can disperse viral particles from rodent excrement over greater distances, expanding the contaminated area and increasing opportunities for human exposure (35). Geographical distribution analysis also indicates that HFRS poses the highest risk of occurrence in China’s mountainous regions with a semi-humid climate (45).

Furthermore, as this study was conducted during the COVID-19 pandemic, the impact of relevant interventions on HFRS incidence must be considered. Pillai et al. (46) noted that incidence levels of respiratory infectious diseases like influenza were underestimated during the COVID-19 pandemic, whereas data integrity for naturally occurring infectious diseases such as HFRS was maintained through continuously operating national infectious disease surveillance information systems (46). Although pandemic control measures—including social distancing and venue restrictions—indirectly altered human activity patterns, Wang et al.’s research indicates that such changes in contact dynamics did not result in significant fluctuations in HFRS incidence rates (47).

This study possesses the following advantages: Firstly, current ecological time-series research on HFRS in China’s Jiaodong Peninsula remains relatively scarce. Most existing studies focus solely on local epidemiological descriptions or surveillance, lacking systematic, multi-city collaborative long-term ecological time-series analysis. Secondly, we identified the key meteorological factors that exert the most significant influence on HFRS among numerous climatic variables. Thirdly, the infection time series for confirmed HFRS cases was based on the date of disease onset, minimizing errors caused by delays in notification. However, some limitations of this study should be noted: Firstly, the collection of HFRS cases depended on passive surveillance across various cities. Potentially leading to underreporting due to limited medical facilities and detection capability. Although this situation has improved remarkably in recent years, underreporting may still occur to some extent. Secondly, this study focuses solely on the lagged effects of meteorological factors on HFRS, without considering the potential influences of hantavirus serotypes, seasonal fluctuations in rodent populations, rodent density, or economic and demographic factors. Finally, the findings of this study are based on data from the Jiaodong Peninsula in China from 2020 to 2024 and may not apply to other regions due to differences in climate and geographical conditions.

## 5 Conclusion

This study systematically elucidates the epidemiological characteristics of meteorological factors on China’s Jiaodong Peninsula, identifying weekly average temperature, weekly average wind speed, weekly average precipitation, and weekly average humidity as key meteorological factors influencing HFRS incidence. It further analyzes the non-linear, interactive effects and complex exposure-lag effects of these meteorological factors on HFRS occurrence between 2020 and 2024. This study provides scientific evidence for understanding the mechanisms by which changes in meteorological factors influence the risk of HFRS, and offers reference for public health authorities to formulate prevention and control strategies for this disease in the context of climate change. Furthermore, the findings suggest that environmental management should be strengthened, with effective measures taken to reduce rodent habitats in order to lower the risk of disease transmission. The model framework and related conclusions developed in this study can be further extended to other HFRS-endemic regions, providing methodological and empirical support for maximizing the control of HFRS outbreaks.

## Data Availability

The data analyzed in this study is subject to the following licenses/restrictions: raw data cannot be made publicly available due to confidentiality and privacy. The data includes sensitive information that could identify patients or compromise their privacy. Moreover, all relevant data have been included in the manuscript and accompanying figures. Readers interested in this study may contact the corresponding author via email if they wish to learn more about the research. Requests to access these datasets should be directed to Qing Duan, sdcdcdq@163.com.

## Footnotes

1
http://data.cma.cn/

## References

[1] Wang N Yin JX. Epidemic process and influencing factors of hemorrhagic fever with renal syndrome: a review. Zhongguo Xue Xi Chong Bing Fang Zhi Za Zhi. (2021) 34:200–3. doi: 10.16250/j.32.1374.2021163, 35537845

[2] Zhang R Mao Z Yang J Liu S Liu Y Qin S . The changing epidemiology of hemorrhagic fever with renal syndrome in southeastern China during 1963-2020: a retrospective analysis of surveillance data. PLoS Negl Trop Dis. (2021) 15:e0009673. doi: 10.1371/journal.pntd.0009673, 34358248 PMC8372920

[3] Avšič-Županc T Saksida A Korva M. Hantavirus infections. Clin Microbiol Infect. (2019) 21:e6–e16. doi: 10.1111/1469-0691.12291 24750436

[4] Taylor SL Schmaljohn CS Williams EP Jonsson CB. Pathogenicity and virulence of rodent-borne Orthohantaviruses. Virulence. (2025) 16:2553784. doi: 10.1080/21505594.2025.2553784, 40878034 PMC12416192

[5] Li H Ling F Zhang S Liu Y Wang C Lin H . Comparison of 19 major infectious diseases during COVID-19 epidemic and previous years in Zhejiang, implications for prevention measures. BMC Infect Dis. (2022) 22:296. doi: 10.1186/s12879-022-07301-w, 35346101 PMC8958816

[6] Jiang H Du H Wang LM Wang PZ Bai XF. Hemorrhagic fever with renal syndrome: pathogenesis and clinical picture. Front Cell Infect Microbiol. (2016) 6:1. doi: 10.3389/fcimb.2016.00001, 26870699 PMC4737898

[7] Tariq M Kim DM. Hemorrhagic fever with renal syndrome: literature review, epidemiology, clinical picture and pathogenesis. Infect Chemother. (2022) 54:1–19. doi: 10.3947/ic.2021.0148, 35384417 PMC8987181

[8] Lee HW. Hemorrhagic fever with renal syndrome in Korea. Rev Infect Dis. (1989) 11:S864–76. doi: 10.1093/clinids/11.Supplement_4.S864 2568676

[9] Vial PA Ferrés M Vial C Klingström J Ahlm C López R . Hantavirus in humans: a review of clinical aspects and management. Lancet Infect Dis. (2023) 23:e371–82. doi: 10.1016/s1473-3099(23)00128-7, 37105214

[10] Sehgal A Mehta S Sahay K Martynova E Rizvanov A Baranwal M . Hemorrhagic fever with renal syndrome in Asia: history, pathogenesis, diagnosis, treatment, and prevention. Viruses. (2023) 15:10.3390/v15020561. doi: 10.3390/v15020561, 36851775 PMC9966805

[11] Zuo SQ Li XJ Wang ZQ Jiang JF Fang LQ Zhang WH . Genetic diversity and the spatio-temporal analyses of hantaviruses in Shandong Province, China. Front Microbiol. (2018) 9:2771. doi: 10.3389/fmicb.2018.02771, 30524397 PMC6257036

[12] Song G. Epidemiological progresses of hemorrhagic fever with renal syndrome in China. Chin Med J. (1999) 112:472–7. 11593522

[13] Dheerasekara K Sumathipala S Muthugala R. Hantavirus infections-treatment and prevention. Curr Treat Options Infect Dis. (2020) 12:410–21. doi: 10.1007/s40506-020-00236-3, 33144850 PMC7594967

[14] Huang X Yin H Yan L Wang X Wang S. Epidemiologic characteristics of haemorrhagic fever with renal syndrome in mainland China from 2006 to 2010. Western Pac Surveill Response J. (2012) 3:1–8. doi: 10.5365/wpsar.2011.2.2.007, 23908902 PMC3729070

[15] Cao B Bai C Wu K La T Su Y Che L . Tracing the future of epidemics: coincident niche distribution of host animals and disease incidence revealed climate-correlated risk shifts of main zoonotic diseases in China. Glob Chang Biol. (2023) 29:3723–46. doi: 10.1111/gcb.16708, 37026556

[16] Chang N Huang W Niu Y Xu Z Gao Y Ye T . Risk of hemorrhagic fever with renal syndrome associated with meteorological factors in diverse epidemic regions: a nationwide longitudinal study in China. Infect Dis Poverty. (2025) 14:3. doi: 10.1186/s40249-024-01272-7, 39815365 PMC11737169

[17] Fang LQ Wang XJ Liang S Li YL Song SX Zhang WY . Spatiotemporal trends and climatic factors of hemorrhagic fever with renal syndrome epidemic in Shandong Province, China. PLoS Negl Trop Dis. (2010) 4:e789. doi: 10.1371/journal.pntd.0000789, 20706629 PMC2919379

[18] Luo Y Zhang L Xu Y Kuai Q Li W Wu Y . Epidemic characteristics and meteorological risk factors of Hemorrhagic fever with renal syndrome in 151 cities in China from 2015 to 2021: retrospective analysis. JMIR Public Health Surveill. (2024) 10:e52221. doi: 10.2196/52221, 38837197 PMC11187512

[19] Xue C Zhang B Li Y Li X Xu C Wang Y. Asymmetric association between meteorological factors and human infections with hemorrhagic fever with renal syndrome: a 16-year ecological trend study in Shaanxi, China. One Health. (2024) 19:100895. doi: 10.1016/j.onehlt.2024.100895, 39318382 PMC11420434

[20] Benedetti A Abrahamowicz M. Using generalized additive models to reduce residual confounding. Stat Med. (2004) 23:3781–801. doi: 10.1002/sim.2073, 15580601

[21] Luo C Qian J Liu Y Lv Q Ma Y Yin F. Long-term air pollution levels modify the relationships between short-term exposure to meteorological factors, air pollution and the incidence of hand, foot and mouth disease in children: a DLNM-based multicity time series study in Sichuan Province, China. BMC Public Health. (2022) 22:1484. doi: 10.1186/s12889-022-13890-7, 35927638 PMC9351082

[22] Gasparrini A Armstrong B Kenward MG. Distributed lag non-linear models. Stat Med. (2010) 29:2224–34. doi: 10.1002/sim.3940, 20812303 PMC2998707

[23] Beck HE Zimmermann NE McVicar TR Vergopolan N Berg A Wood EF. Present and future Köppen-Geiger climate classification maps at 1-km resolution. Sci Data. (2018) 5:180214. doi: 10.1038/sdata.2018.214, 30375988 PMC6207062

[24] Liu Y Zhao H. Variable importance-weighted random forests. Quant Biol. (2017) 5:338–51. doi: 10.1007/s40484-017-0121-6 30034909 PMC6051549

[25] Li C Zhao Q Zhao Z Liu Q Ma W. The association between tropical cyclones and dengue fever in the Pearl River Delta, China during 2013-2018: a time-stratified case-crossover study. PLoS Negl Trop Dis. (2021) 15:e0009776. doi: 10.1371/journal.pntd.0009776, 34499666 PMC8454958

[26] Yue ZH Han X Wei YM Cai YN Han ZY Zhang YB . Study of prediction of hemorrhagic fever with renal syndrome incidence in Hebei Province based on generalized additive model. Zhonghua Liu Xing Bing Xue Za Zhi. (2025) 46:418–22. doi: 10.3760/cma.j.cn112338-20240813-00499, 40113392

[27] Liao J Yu S Yang F Yang M Hu Y Zhang J. Short-term effects of climatic variables on hand, foot, and mouth disease in mainland China, 2008-2013: a multilevel spatial Poisson regression model accounting for overdispersion. PLoS One. (2016) 11:e0147054. doi: 10.1371/journal.pone.0147054, 26808311 PMC4726563

[28] Wang P Zhang X Hashizume M Goggins WB Luo C. A systematic review on lagged associations in climate-health studies. Int J Epidemiol. (2021) 50:1199–212. doi: 10.1093/ije/dyaa286, 33448301

[29] Li CP Cui Z Li SL Magalhaes RJ Wang BL Zhang C . Association between hemorrhagic fever with renal syndrome epidemic and climate factors in Heilongjiang Province, China. Am J Trop Med Hyg. (2013) 89:1006–12. doi: 10.4269/ajtmh.12-0473, 24019443 PMC3820312

[30] Gasparrini A. Distributed lag linear and non-linear models in R: the package dlnm. J Stat Softw. (2011) 43:1–20. doi: 10.18637/jss.v043.i08, 22003319 PMC3191524

[31] Sun W Liu X Li W Mao Z Sun J Lu L. Effects and interaction of meteorological factors on hemorrhagic fever with renal syndrome incidence in Huludao City, northeastern China, 2007-2018. PLoS Negl Trop Dis. (2021) 15:e0009217. doi: 10.1371/journal.pntd.0009217, 33764984 PMC7993601

[32] Chen Y Hou W Dong J. Time series analyses based on the joint lagged effect analysis of pollution and meteorological factors of hemorrhagic fever with renal syndrome and the construction of prediction model. PLoS Negl Trop Dis. (2023) 17:e0010806. doi: 10.1371/journal.pntd.0010806, 37486953 PMC10399869

[33] Zheng L Gao Q Yu S Chen Y Shi Y Sun M . Using empirical dynamic modeling to identify the impact of meteorological factors on hemorrhagic fever with renal syndrome in Weifang, northeastern China, from 2011 to 2020. PLoS Negl Trop Dis. (2024) 18:e0012151. doi: 10.1371/journal.pntd.0012151, 38843297 PMC11185475

[34] Cao L Huo X Xiang J Lu L Liu X Song X . Interactions and marginal effects of meteorological factors on haemorrhagic fever with renal syndrome in different climate zones: evidence from 254 cities of China. Sci Total Environ. (2020) 721:137564. doi: 10.1016/j.scitotenv.2020.137564, 32169635

[35] Luo Y Lv H Yan H Zhu C Ai L Li W . Meteorological change and hemorrhagic fever with renal syndrome epidemic in China, 2004-2018. Sci Rep. (2022) 12:20037. doi: 10.1038/s41598-022-23945-9, 36414682 PMC9681842

[36] Bai XH Peng C Jiang T Hu ZM Huang DS Guan P. Distribution of geographical scale, data aggregation unit and period in the correlation analysis between temperature and incidence of HFRS in mainland China: a systematic review of 27 ecological studies. PLoS Negl Trop Dis. (2019) 13:e0007688. doi: 10.1371/journal.pntd.0007688, 31425512 PMC6715292

[37] Xu DL Xu MM Wang DH. Effect of temperature on antioxidant defense and innate immunity in Brandt's voles. Zool Res. (2019) 40:305–16. doi: 10.24272/j.issn.2095-8137.2019.045, 31310064 PMC6680122

[38] Ferro I Bellomo CM López W Coelho R Alonso D Bruno A . Hantavirus pulmonary syndrome outbreaks associated with climate variability in northwestern Argentina, 1997-2017. PLoS Negl Trop Dis. (2020) 14:e0008786. doi: 10.1371/journal.pntd.0008786, 33253144 PMC7728390

[39] Tian H Stenseth NC. The ecological dynamics of hantavirus diseases: from environmental variability to disease prevention largely based on data from China. PLoS Negl Trop Dis. (2019) 13:e0006901. doi: 10.1371/journal.pntd.0006901, 30789905 PMC6383869

[40] Zhang WY Wang LY Liu YX Yin WW Hu WB Magalhaes RJ . Spatiotemporal transmission dynamics of hemorrhagic fever with renal syndrome in China, 2005-2012. PLoS Negl Trop Dis. (2014) 8:e3344. doi: 10.1371/journal.pntd.0003344, 25412324 PMC4239011

[41] Skanata A Spagnolo F Metz M Smyth DS Dennehy JJ. Humidity reduces rapid and distant airborne dispersal of viable viral particles in classroom settings. Environ Sci Technol Lett. (2022) 9:632–7. doi: 10.1021/acs.estlett.2c00243, 35937034 PMC9344459

[42] Ji H Li K Shang M Wang Z Liu Q. The 2016 severe floods and incidence of Hemorrhagic fever with renal syndrome in the Yangtze River basin. JAMA Netw Open. (2024) 7:e2429682. doi: 10.1001/jamanetworkopen.2024.29682, 39172449 PMC11342140

[43] Hardestam J Simon M Hedlund KO Vaheri A Klingström J Lundkvist A. Ex vivo stability of the rodent-borne Hantaan virus in comparison to that of arthropod-borne members of the Bunyaviridae family. Appl Environ Microbiol. (2007) 73:2547–51. doi: 10.1128/aem.02869-06, 17337567 PMC1855600

[44] Zhu LL Li YP Lu L Li SJ Ren HY. Spatial heterogeneity and influencing factors of HFRS epidemics in rural and urban areas: a study in Guanzhong plain of Shaanxi Province, China. Biomed Environ Sci. (2022) 35:1012–24. doi: 10.3967/bes2022.130, 36443254

[45] Wang Y Duan Q Pang B Tian X Ma J Ma W . Assessing the relationship between climate variables and Hemorrhagic fever with renal syndrome transmission in eastern China: a multi-cities time series study. Transbound Emerg Dis. (2023) 2023:1–9. doi: 10.1155/2023/5572334, 40303818 PMC12016773

[46] Sabeena S Ravishankar N Robin S. The impact of COVID-19 pandemic on influenza surveillance: a systematic review and meta-analysis. Indian J Public Health. (2022) 66:458–65. doi: 10.4103/ijph.ijph_926_22, 37039174

[47] Wang Y Qing S Lan X Li L Zhou P Xi Y . Evaluating the long-term impact of COVID-19-associated public health interventions on zoonotic and vector-borne diseases in China: an interrupted time series analysis. J Transl Med. (2024) 22:81. doi: 10.1186/s12967-024-04855-y, 38245788 PMC10799468

