# Supplementary material for: Meteorological drivers of hemorrhagic fever with renal syndrome in China’s Jiaodong Peninsula: an ecological time-series study from 2020 to 2024
Source: Front Public Health. 2025 Dec 16;13:1743845. doi: 10.3389/fpubh.2025.1743845 (PMC12748265; doi:10.3389/fpubh.2025.1743845)
Supplement: Supplementary file 1 [file Data_Sheet_1.pdf]

Meteorological drivers of hemorrhagic fever with renal syndrome in China's Jiaodong Peninsula: an ecological time-series study from 2020 to 2024

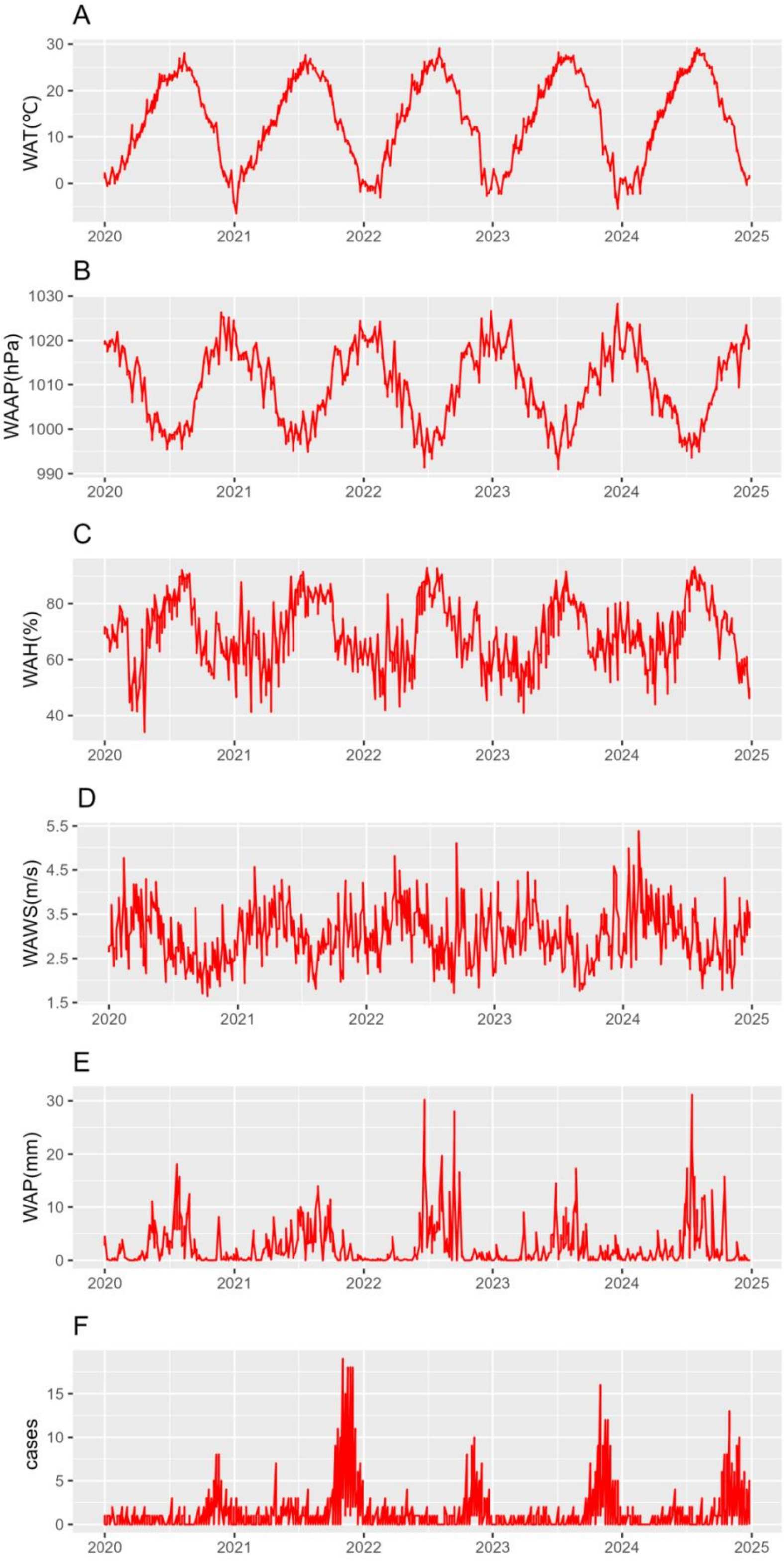

Supplementary Figure 1 | Weekly distribution of HFRS cases and meteorological factors in the Jiaodong Peninsula, 2020-2024.
